# A Glimpse of Programmed Cell Death Among Bacteria, Animals, and Plants

**DOI:** 10.3389/fcell.2021.790117

**Published:** 2022-02-10

**Authors:** Jun Zhuang, Li Xie, Luping Zheng

**Affiliations:** ^1^ State Key Laboratory of Ecological Pest Control for Fujian and Taiwan Crops, Fujian Agriculture and Forestry University, Fuzhou, China; ^2^ Institute of Plant Virology, Fujian Agriculture and Forestry University, Fuzhou, China

**Keywords:** pyroptosis, necroptosis, apoptosis, hypersensitive cell death response, ferroptosis, bacterial PCD

## Abstract

Programmed cell death (PCD) in animals mainly refers to lytic and non-lytic forms. Disruption and integrity of the plasma membrane are considered as hallmarks of lytic and apoptotic cell death, respectively. These lytic cell death programs can prevent the hosts from microbial pathogens. The key to our understanding of these cases is pattern recognition receptors, such as TLRs in animals and LRR-RLKs in plants, and nod-like receptors (NLRs). Herein, we emphatically discuss the biochemical and structural studies that have clarified the anti-apoptotic and pro-apoptotic functions of Bcl-2 family proteins during intrinsic apoptosis and how caspase-8 among apoptosis, necroptosis, and pyroptosis sets the switchable threshold and integrates innate immune signaling, and that have compared the similarity and distinctness of the apoptosome, necroptosome, and inflammasome. We recapitulate that the necroptotic MLKL pore, pyroptotic gasdermin pore, HR-inducing resistosome, and mitochondrial Bcl-2 family all can form ion channels, which all directly boost membrane disruption. Comparing the conservation and unique aspects of PCD including ferrroptosis among bacteria, animals, and plants, the commonly shared immune domains including TIR-like, gasdermin-like, caspase-like, and MLKL/CC-like domains act as arsenal modules to restructure the diverse architecture to commit PCD suicide upon stresses/stimuli for host community.

## Introduction

**FIGURE 1:**
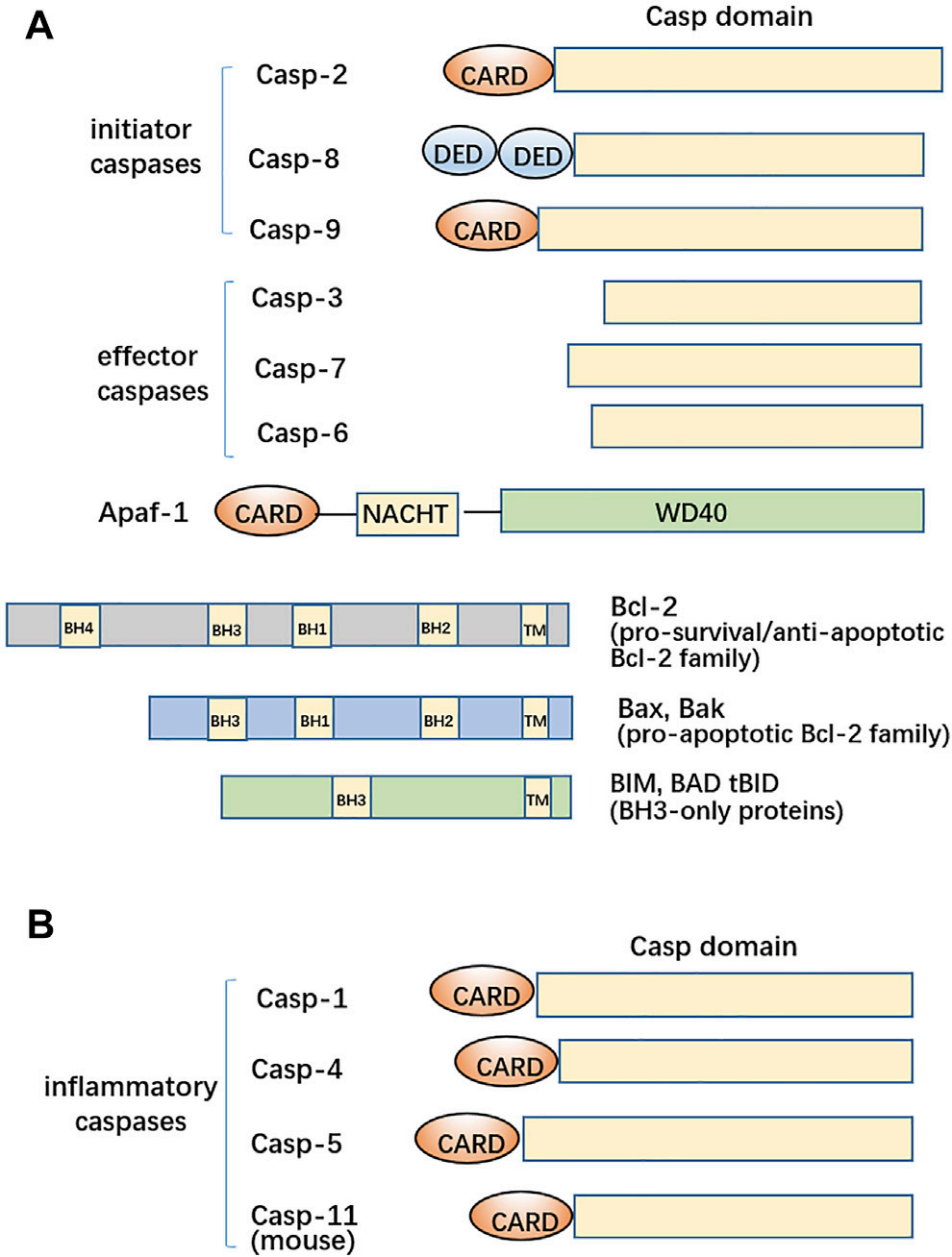
Structural features of caspases, Apaf-1, and the Bcl-2 family. **(A)** Master initiator caspases, caspase-2, caspase-8, and caspase-9, are characterized by N-terminal domain(s) including DED or CARD. The effector caspases have caspase-3, caspase-6, and caspase-7 for apoptosis. Apaf-1 is a main scaffold protein for recruitment of Cyt c and caspase-9. Bcl-2 itself plays anti-apoptotic roles whereas the Bax/Bak members of the Bcl-2 family are pro-apoptotic. BH3-only protein is an intrinsic apoptosis initiator. **(B)** Another clade contains inflammatory caspases, including human caspase-1, -4, and -5 and mouse caspase-11, have the N-terminal CARD domains.

Programmed cell death (PCD) is clearly characterized in animals and contains several types. Of PCDs, apoptosis is required for tissue development, maintenance of the homeostasis of proliferating cells, and multicellular morphogenesis in plants and animals. The conceptual proposal of apoptosis started from 1965 (Kerr, 1965). Australian scientists discovered that some scattered dead cells from the liver parenchyma were present when observed under an electron microscope after ligation of the rat portal vein. The lysosomes from these cells seemed not to be damaged and were kept in an intact situation. These cells, featured by morphological shrinkage and chromatin aggregations, fall off from their surrounding tissues and were ultimately engulfed. Kerr and other three scientists formally put forward the concept of apoptosis in 1972 (Kerr et al., 1972). The molecular progresses on apoptosis *per se* began with a good model organism *Caenorhabditis elegans*. Sydney Brenner first determined the *C. elegans* cell development lineage (Brenner, 1973). John Sulston discovered the specific cell division and differentiation during the nematode developmental process and identified that nematode apoptosis is dictated by alternate gene expressions. Robert Horvitz found more than 20 genes regulating apoptosis (Lettre and Hengartner, 2006; Ellis and Horvitz, 1986). These come in (at least) two distinct flavors, containing either ones responsible for initiating or executing cell death or others involved in inhibition of cell death. The four genes that regulate all somatic cell deaths in *C. elegans* are *CED-3*, *CED-4*, *CED-9*, and *EGL-1* genes. CED-9 is an anti-apoptotic Bcl-2 homolog with four Bcl-2 homology (BH) domains, whereas EGL-1 acts as a pro-apoptotic BH3-only domain protein (Hengartner et al., 1992) (Figure 1A); CED-3 and CED-4 are pro-apoptotic (Lettre and Hengartner, 2006). CED-3 is homologous to mammalian caspases (cysteinyl aspartic acid–specific proteases) formerly known as interleukin-1B–converting enzyme in animals (Yuan et al., 1993), and CED-4 is an adaptor protein that is orthologous to mammalian apoptotic protease–activating factor-1 (Apaf-1) being the main scaffold protein of apoptosome for caspase-9 activation in the intrinsic pathway (Lettre and Hengartner, 2006) (Figure 1A). Although the three conserved gene-encoding proteins regulate apoptosis in animals, no corresponding orthologous proteins have been found in plants.

### Mammalian Apoptosis Mediated by Caspases

**FIGURE 2:**
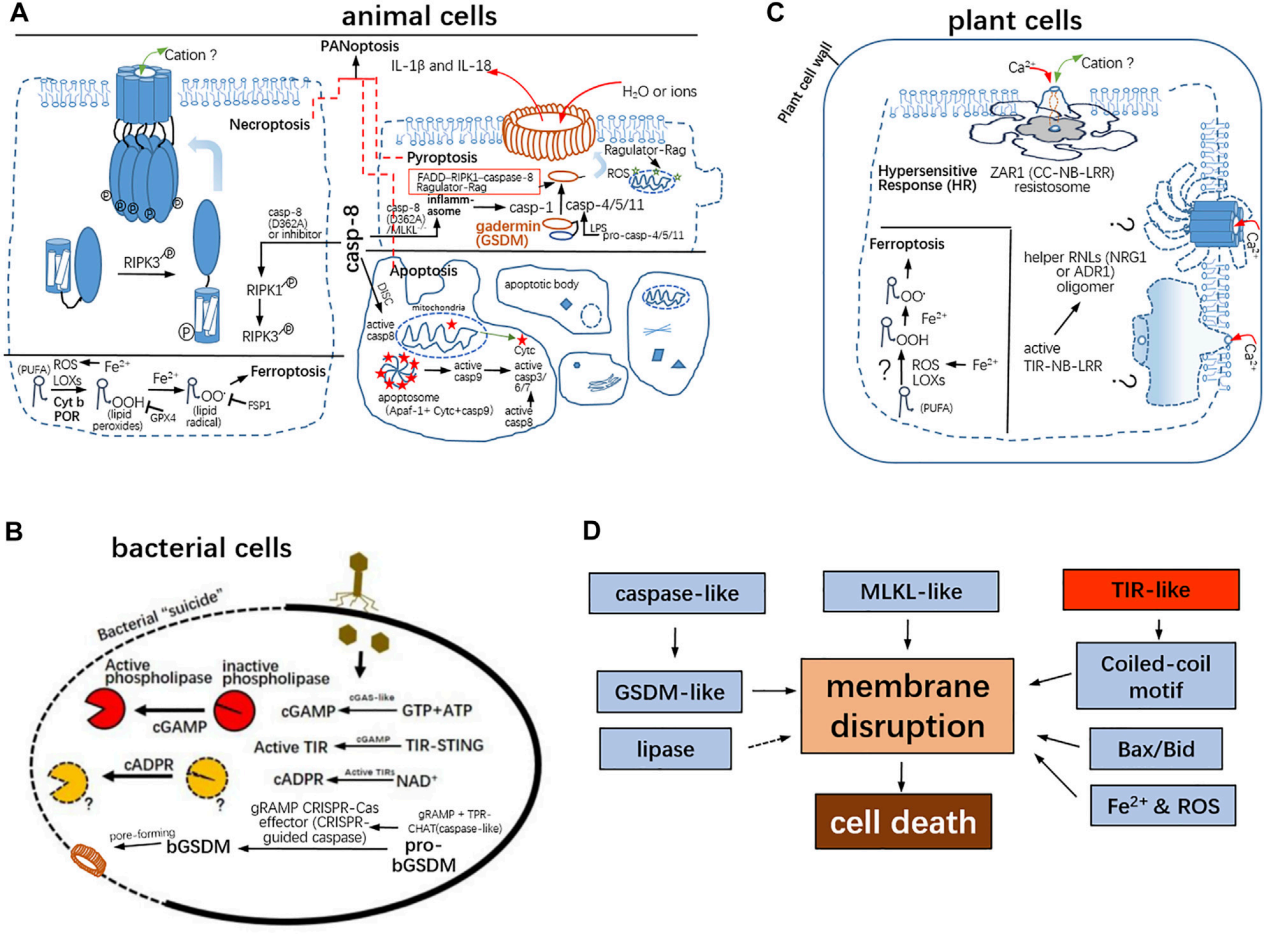
Commonality of various PCDs among bacteria, animals, and plants. **(A)** Necroptosis is mediated by RIPK3-phosphorylated MLKL. The oligomerization of the 4-helice bundle in the N-terminal region of MLKL should form a cation channel in the plasma membrane and lead to disruption of membrane integrity. Inflammatory caspases were activated by canonical and/or non-canonical inflammosomes. The active inflammatory caspases can cleave gasdermins. The resulting N-gasdermins insert the plasma membrane and form a membrane pore with 18–21 nm in inner diameter. The gasdermin pore prefers the release of IL-1β and IL-18. Apoptosis is a non-lytic cell death. In the instinct pathway, the initiator caspase-9 is activated by the apoptosome consisting of apaf-1, Cyt c, and caspase-9; caspase-8 is activated by the death-inducing signaling complex (DISC) for extrinsic apoptosis. Caspase-8 is the molecular switch for apoptosis, for necroptosis, and pyroptosis. Ferroptosis is required for iron and is mediated by LOXs and ROS and POR and Cyt b. The lipid peroxides and lipid radicals are capable of being sequestered by GPX4 and FSP1, respectively. Upon infection, PANoptosis co-featured by pyroptosis, apoptosis, and necroptosis is present as well. **(B)** The bacterial gasdermins are conserved and commit cell death *via* pore-forming as well. cGAMP as the elicitor activates the phospholipase, which perturb membrane integrity and result in cell death. gRAMP, the giant repeat–associated mysterious protein from CRISPR-Cas type III effectors; TPR-CHAT, caspase HetF associated with the tetratricopeptide repeat. proteases **(C)** Resistance (R) protein-mediated HR and ferroptosis-like in the plant cell. The R protein ZAR1 (CC-NBS-LRR) with RKS1 and PBL2 ^UMP^ ligands form the pentameric complex. The funnel-like channel (∼0.5 nm in narrowest diameter) is a non-selective, Ca^2+^ influx, cation channel. Likewise, helper NLRs required for TLR (TIR-NB-LRR)-mediated HR constitute a Ca^2+^ influx, cation channel. But the definitive 3D models of helper NLRs remain elusive; other CNL resistosomes may be cation channels, but their 3D structures are not be determined. And, ferroptosis-like cell death was also observed in plant cells. **(D)** In total, GSDM and MLKL domains directly target the plasma membrane; the Bcl-2 family is responsible for mitochondrial membrane damages; and TIR-like sense and monitor energy deficit to produce a second messenger for downstream signaling.

During the process of apoptosis, the central hub is the activation of caspases. Human caspases have 11 members and are categorized into 3 subclasses (Figures 1A,B). Different clades largely correspond to distinct physiological functions. Not all caspases are involved in apoptotic regulation, *albeit* caspases dictate the destiny of apoptotic cells. Caspase-2, -8, -9, and -10 are involved in the initiation of apoptosis (as initiators). Upon dimerization, procaspase-2 becomes active and can process cytosolic Bid to trigger the release of Cyt c (Baliga et al., 2004; Guo et al., 2002); caspase-8 and caspase-9 individually initiate the extrinsic and intrinsic mammalian apoptosis (Chai and Shi, 2014), or caspase-8 has an N-terminal tandem death effector domain (DED) and is coordinated with the death receptor TNFR for perception of extracellular death signals. Hence, recruitment of caspase-8 forms a death-inducing signaling complex (DISC) and then activates caspase-8 (Schleich et al., 2013). The Apaf-1 apoptosome assembles into a heptameric wheel-like complex with cytochrome c (Cyt c) and caspase-9 having the N-terminal caspase recruitment domain (CARD) (Li et al., 1998). The dome of apoptosome was identified to be the oligomer of CARDs from Apaf-1 and caspase-9 (7:3-4 stoichiometry); then, procaspase-9 undergoes conversion into caspase-9 (Figure 2A). However, the DARK-DRONC apoptosome (8:8 stoichiometry) complex found in flies and the *C. elegans* octameric CED-4 apoptosome interacting with CED-3 (8:2 stoichiometry) does not require cytochrome c to assemble, as it does in humans. This activating ligand Cyt c for Apaf-1 can be released from the perforated mitochondria by pro-death Bad/Bax, which is being initiated *via* activation of Bid by caspase-8 (Li et al., 1998; Antignani and Youle, 2006). The plasma membrane of apoptotic cells keeps basically intact; however, the perforated mitochondrial outer membrane by Bax/Bak alternates the mitochondrial outer membrane permeabilization (MOMP) during intrinsic apoptosis. Then, endonuclease G (Endo G) is released from the disrupted mitochondria and enters the nucleus, resulting in DNA cleavage to form DNA ladders, which can also be contributed by DNase *γ* (Li et al., 2001; Shiokawa et al., 2002). Additionally, the active caspase-8 sequentially activates effector caspase(s) (Stennicke et al., 1998). The activated caspase-3 and -7 have similar cleavage profiles of substrates. The degradations of poly (ADP-ribose) polymerase (PARP) and DNA fragmentation factor-45 (DFF-45) by caspase-3 and caspase-7 give rise to failures in DNA repair and initiation of DNA degradation (Jänicke et al., 1998; Tang and Kidd, 1998; Wride et al., 1999). The lamin A critical for nuclear architecture acts as the substrate of activated caspase-6 (Ruchaud et al., 2002). Degraded lamin A and other cellular skeletal proteins lead to cellular shrinkages and chromatin condensations. However, the cytokines cannot leak out from apoptotic cells to the bystander cells due to the integrity of the plasma membrane during apoptosis genesis. Therefore, apoptosis without the release of cytokines cannot sequentially induce inflammation.

### The Emerging Roles of Mammalian Bcl-2 Family Proteins in Apoptosis

The protein members of the B-cell lymphoma 2 (Bcl-2) family residing at the outer mitochondrial membrane may be classified into three functionally and structurally distinct subgroups, such as BH3 (the Bcl-2 homology 3)-only proteins (which communicate signals to initiate intrinsic apoptosis), the Bcl-2 itself as the pro-survival cell guardian, and the pro-apoptotic effector proteins BAX (Bcl-2–associated X protein) and BAK (Bcl-2 antagonist/killer) (Lindsten et al., 2000; Czabotar et al., 2014; Ke et al., 2012; Naim and Kaufmann, 2020) (Figure 1A). The anti- and pro-apoptotic ones both balance the mitochondrial membrane potential, and their interplay sets the apoptotic threshold in the mitochondrial outer membrane. With respect to the BH3-only protein, there are activator BH3-only proteins including BIM and the truncated form of BID (tBID) that can directly bind and activate BAX or BAK and sensitizer BH3-only proteins, such as BAD, which indirectly activate BAX or BAK by neutralizing pro-survival Bcl-2 family members (Czabotar et al., 2014). Overexpressed BH3-only proteins, particularly those (BIM, tBID, and PUMA) that target all pro-survival Bcl-2 family members, can trigger apoptosis. Diverse cell types from *Bax* ^*−/−*^ *Bak* ^*−/−*^ mice are completely resistant to multiple apoptotic stimuli, including the enforced expression of BH3-only proteins (Wei et al., 2001; Cheng et al., 2001; Zong et al., 2001) and are required for normal tissue development (Rathmell et al., 2002; Mason et al., 2013). Thus, the BH3-only proteins function upstream of BAX and BAK (Czabotar et al., 2014) and cannot cause cell death unless BAX or BAK is present. In transgenic mice, all of the pro-survival Bcl-2 family members endow much cell type resistance against diverse apoptotic stimuli. The apoptosis of autoreactive *BIM* ^−/−^ B cells and T cells negatively regulated by Bcl-2 act as one important checkpoint for preventing autoimmune kidney disease that resembles human systemic lupus erythematosus. BH3-only proteins or BH3-mimicking small molecules (BH3 mimetics) might promote apoptosis and improve the cancer therapy effect. Accordingly, the Bcl-2 overexpression in B cells of mice, loss of BIM, or loss of both BAX and BAK can provoke a fatal autoimmune kidney disease and might improve the treatment of diverse types of cancer (Adams and Cory, 2018).

### MLKL-Mediated Necroptosis in Mammalian Cells

As known, non-lytic apoptosis is a non-inflammatory form of cell death. On the contrary, necroptosis and pyroptosis belong to lytic PCDs along with inflammatory exudates but differ for distinguishably lytic phenotypes. The necroptotic feature corresponds to cellular explosion, and pyroptosis is characterized by osmotic swelling/balloon-like protrusions. Necroptotic stimuli (including Z-DNA, Z-DNA binding protein 1 (ZBP1), and TNF-α) initiate a supramolecular organizing center (SMOC), called the necrosome RIPK1-RIPK3 core, as a hetero-amyloid signaling complex to perform autophosphorylations (Zhang, et al., 2009; Sun et al., 2012; Sun and Wang, 2014) (Figure 2A). The activated RIPK3 phosphorylates the necroptosis effector, as a pseudokinase—mixed lineage kinase domain-like (MLKL). And, the phosphorylated MLKLs undergo conformational alternation and initiatively bind with IP6 and recognize the anionic phospholipids (such as phosphatidylinositol-4-phosphate (PtIns4P)) of the inner leaflet of the plasma membrane, finally oligomerize and perforate the plasma membrane to form a non-selective cation channel (Huang et al., 2017; Su et al., 2014; Wang et al., 2014) (Figure 2A). Necroptosis would occur upon death of stimuli when caspase-8 is inactive after genetic depletion or chemical inhibition by the Z-VAD-FMK inhibitor (He, et al., 2009) (Figure 2A). In the more complex necroptotic pathway, tumor necrosis factor receptor 1 (TNFR1), toll-like receptor 3 (TLR3)–TRIF, and TLR4–TRIF signal *via* RIPK1 to activate NF-κB, but RIPK1 is not required for the TRIF-type I IFN response (Fitzgerald and Kagan, 2020).

### Gasdermin-Mediated Pyroptosis in Animals and Bacterial Cells

Like apoptosomes and necroptosomes, pyroptotic inflammasomes are responsible for the activation of inflammatory caspase-1 (Ding and Shao, 2017). How are the other inflammatory caspase-4/5/11 activated upon bacterial infections? Shao Lab identified that caspase-4/5/11 can directly recognize the cytosolic lipopolysaccharides (LPS) to aggregate into non-canonical inflammasomes and commit self-cleavage to form active caspases (Shi et al., 2014). Once activated, caspase-1, 4/5/11 are capable of cleaving gasdermin D (GSDMD), being a bipartite protein whose amino-terminal and carboxy-terminal domains are connected by a linker and the free *N*-terminal fragment of GSDMD to induce 31∼34-fold symmetry gasdermin pore forming and pyroptosis (Shi et al., 2015; Ding et al., 2016; Ding and Shao, 2017; Ruan et al., 2018; Xia et al., 2021) (Figure 2A). The mature forms of pro–interleukin-1β (IL-1β) and pro–IL-18 processed by caspase-1 are feasible to outflow through the GSDMD pore (∼21 nm in internal diameter), while large amounts of sodium and water enter, and increasing expansion of pores finally lead to osmotic swelling and cell death (Xia et al., 2021) (Figure 2A).

The gasdermin subfamily has six members GSDMA-GASDME and GSDMF (DFNB59, also called PJVK) in humans. GSDMF is highly similar to GSDME, and its mutation is associated with autosomal recessive deafness (Broz et al., 2020). Different gasdermin members are activated by different protease(s) including caspase(s) or other proteases. The activation of GSDME was licensed to caspase-3, and activated gasdermin E assemblies (pore rings) have 26∼28-fold symmetry and may drive chemotherapy-induced pyroptosis (Wang et al., 2017; Broz et al., 2020; Zhang et al., 2020; Xia et al., 2021). And, gasdermin B can also be functionally cleaved by granzyme(s) from lymphocytes and NK cells for activation (Zhou et al., 2020). Apart from targeting the plasma membrane, active gasdermin B prefers to bind bacterial phospholipids and has strong bactericidal activities rather than cellular toxicity for NK cells (Hansen et al., 2021).

Importantly, oligomerization of the released N-lobe of gasdermins is a requisite for pyroptotic cell death. Pyroptosis is defined as gasdermin-mediated programmed necrotic cell death (Broz et al., 2020). As known, pyroptosis accompanies mitochondrial damages. And, the mitochondrial tricarboxylic acid (TCA) cycle metabolites (namely, fumarate and its derivatives) can modify a reactive cysteine of GSDMD by succination, which results in significantly decreased cleavage and pore formation (Humphries et al., 2020). Nevertheless, GSDMD cleavage by proteases appears to be not equivalent to pore formation. Recent studies identified that the lysosome-locating Ragulator-Rag complex involved in the mTORC1 pathway maintaining metabolic homeostasis may control the pore formation of gasdermins in the plasma membrane and/or serve as a scaffold for the activation of the FADD–RIPK1–caspase-8 complex to induce pyroptosis (Evavold et al., 2021; Zheng et al., 2021) (Figure 2A). The mTORC1 sensitize and control mitochondrial dysfunction and promote mitochondrial ROS generation, which boost gasdermin oligomerization *in vivo* (Evavold et al., 2021). The Rag-Ragulator complex surveils both metabolism and infection to act as a molecular hub dictating the living or death fate of infected cells (Zheng et al., 2021).

During the last stage of pyroptotic cell death, the plasma membrane rupture (PMR) usually occurs in dying cells. PMR previously is considered as a passive process following pore formation. But Kayagaki et al. (2021) recently identified that PMR may be a positive event mediated by the protein NINJ1, which is a 16-kDa protein with two transmembrane motifs and juxtaposed to the plasma membrane with both termini outside the cytoplasm. In addition to pyroptosis, other programmed lytic cell deaths all undergo PMR as well. Therefore, NINJ1 may act downstream of the formations of the GSDMD pore or MLML channel to elicit PMR.

Apart from mammalian gasdermins, the only CsGSDME from aquatic teleost *Cynoglossus semilaevis* can be cleaved by Cscaspase-1, -3, and -7 to elicit pyroptosis (Jiang et al., 2019). Additionally, the GSDME homolog also exists in the marine invertebrate coral *Orbicella faveolata*, and Ofcaspase-3 is capable of cleaving OfGSDME to induce pyroptosis upon the infection by the bacterial pathogen *Vibrio coralliilyticus* (Jiang S et al., 2020). These important findings of GSDME-mediated pyroptosis in aquatic animals shed light on the activation mode of gasdermin during pyroptosis and broaden the evolutionary insights into pyroptosis-related immunological stresses upon bacterial invasions. In contrast to animals’ gasdermins, the uncharacterized proteins with predicted homology to gasdermin domains were identified after bioinformatical analyses of bacterial anti-phage defense islands. The majority are encoded adjacent to one or more genes with a predicted protease domain through examining the genomic neighborhood of bacterial gasdermin-likes (Johnson et al., 2022). Some of the GSDM-associated proteases are fused to repeat domains including leucine-rich repeats, tetratricopeptide repeats, WD40 repeats, or NACHT domains frequently involved in prokaryotic samples. In addition, the gRAMP CRISPR-Cas effector is an RNA endonuclease complex with a caspase-like peptidase (van Beljouw et al., 2021), regardless of the structure and substrate specificity of bacterial caspase-like proteases temporarily named ‘‘orthocaspases’’ (Minina et al., 2020). While breaking of viral RNAs is inadequate to escape infections, bacteria would switch on suicide as a consequence of activation of caspase-likes by sensing viral RNAs. Another recent report claimed that the activated *Runella* gasdermin-likes after removal of the short C-terminal region (about 20 AAs) by the associated caspase-like protease (or a certain orthocaspase) has the capability to form mesh-like membrane pores (average 28 nm in inner diameter) and displays bactericidal activity *via* non-selective leakage (Johnson et al., 2022) (Figure 2B), although how caspase-like protease becomes active was not documented. Collectively, it uncovered that the conserved gasdermin-like pore is an ancient conduit for the cellular content efflux in prokaryotes and eukaryotes.

### The Switch of Apoptosis, Necroptosis, and Pyroptosis by Caspase-8

In response to influenza A virus (IAV) infection, the induced pro-death complex encompasses a plethora of proteins: RIPK1, apoptosis-associated speck-like protein containing a caspase recruitment domain (ASC), nucleotide-binding oligomerization domain NOD-like receptor pyrin domain-containing 3 (NLRP3), and caspase-8, RIPK3, ZBP1, and caspase-1 (Samir et al., 2020). In addition, the AIM2 sensitizing dsDNA sense double-stranded DNA (dsDNA) forms the inflammasome being an important sentinel of the innate immune defense and has essential roles in development of infectious diseases. However, AIM2 beyond its canonical role in inflammasome formation and observed pyroptosis cannot explain the outcome resulted from the AIM2 inflammasome. During infections by dsDNA herpes simplex virus 1 (HSV1) and the Gram-negative bacterium *Francisella novicida*, AIM2, pyrin, and ZBP1 were constituents of a large multiplex complex concomitant with ASC,caspase-1, caspase-8, RIPK3, RIPK1, and FADD, that led to PANoptosis, an inflammatory cell death pluralized by apoptosis, pyroptosis, and necroptosis (Lee et al., 2021) (Figure 2A). The confluence of critical molecules for apoptosis, pyroptosis, and necroptosis could explain why the different types of cell death can exchange under certain conditions (Schwarzer et al., 2020). Caspase-8 is the initiator caspase of extrinsic apoptosis and cleaves RIPK1/3 to restrict necroptosis (Frank and Vince, 2019). Therefore, caspase-8 deficiency in mice causes embryonic lethality which can be rescued by deletion of either RIPK3 or MLKL (Fritsch et al., 2019; Newton et al., 2019). MLKL deficiency rescues the cardiovascular defect phenotype but unexpectedly causes necroptosis-independent death. When necroptosis is blocked, the expression of non-catalytic caspase-8 triggered the formation of ASC-associated inflammasomes and resulted in pyroptosis in mice. Genetic analyses confirmed that caspase-8 serves as the molecular switch for hierarchical activation of apoptotic, necroptotic, and pyroptotic signaling pathways (Orning et al., 2018; Fritsch et al., 2019; Newton et al., 2019) (Figure 2A).

### Animal and Plant Bcl-2–Associated Athanogene Proteins in Cell Death Regulation and Stress Responses

The extrinsic apoptosis pathway and other types of PCD are orchestrated by caspase-8, whereas MOMP during intrinsic apoptosis is positively and negatively regulated by Bcl-2 family proteins. To identify Bcl-2 partner(s), the Bcl-2–associated athanogene (BAG) family genes were initially found *via* a yeast two-hybrid screening (Kabbage and Dickman, 2008). The *BAG1* gene was shown to enhance the anti-apoptotic activity of Bcl-2, which seemed to be indicative of its involvement in the apoptotic pathway(s) (Takayama et al., 1995; Brive et al., 2001; Takayama and Reed, 2001). The BAG family is a phylogenetically conserved group of proteins with orthologues widely across organisms from plants to metazoans including humans. The C-terminal BAG domain (BD) from all BAG proteins directly interact with the heat shock protein 70 (HSP70) chaperone (Takayama and Reed, 2001). The BAG proteins serve as co-chaperones that function as molecular switches associating with HSP70 and other substrates and maintain protein homeostasis and modulate cell death. In fact, BAG1 itself functions as a substrate of E3 ligase (i.e., the C terminus of HSC70-interacting protein (CHIP)), and the formation of the BAG1-CHIP ternary complex targets proteins for degradation (Kabbage et al., 2017). Reversely, BAG2 associating with CHIP inhibits the E3 ligase activity. BAG3 has roles in protein quality control to sustain cell survival and was indicative of the antagonistic effect against chemotherapy (Behl 2016). BAG4 has been considered to act as a negative regulator of the TNF superfamily. BAG5 has been relevant to neurodegeneration (such as Parkinson’s disease) and was discovered to suppress both parkin E3 ligase and HSP70 chaperone activities (Kalia et al., 2004). BAG6 ablation might contribute to increased lethality and severe developmental abnormality in various organs (Kabbage et al., 2017).

*Arabidopsis* BAG proteins may be categorized into two sub-groups according to their featured domain: AtBAG1–4 having a UBL motif similar to human BAG1 besides the BD, and AtBAG5–7 containing a calmodulin (CaM)–binding motif nearby the BD. The AtBAG1–3 keeps functionally unknown. BAG4 binds to HSP70 chaperones and is related to cell death inhibition upon abiotic stress. AtBAG5 constitutes a complex with CaM/HSC70 and is involved in plant senescence (Kabbage et al., 2017). AtBAG6 is functionally activated through aspartyl protease processing and coordinates with chitin perception to inducible autophagy (Kang et al., 2006). The ER-locating AtBAG7, as an essential component of the unfolded protein response, recognizes the molecular chaperone BIP2. Upon ER stresses, AtBAG7 can translocate to the nucleus, where it interplays with the transcription factor WRKY29 related to stress response and/or immunity (Li et al., 2017). Due to BAGs’ association with HSP70 partially and their multiplex targets, the conservation of BAG molecular regulations and contributive properties in immunity-associated cell death was discovered in plants and animals.

### Ferroptosis in Animal and Plant Cells

Along with the discovery of apoptosis, necroptosis, pyroptosis, and immune cell death, ferroptosis dependent of iron was proposed in 2012 (Dixon et al., 2012). Ferroptosis is characterized by the peroxidation of polyunsaturated fatty acids (PUFAs) from membrane lipids by lipoxygenases (LOXs) being non-heme iron oxidases and reactive oxygen species (ROS) from the Fe^2+^-directed Fenton reaction (Yang et al., 2016). Recently, lipid peroxidation during ferroptosis may be mainly catalyzed by oxidoreductases POR and cytochrome b5 reductase 1 (CYB5R1) other than LOXs (Yan et al., 2021). Importantly, ferroptosis can be hindered by glutathione peroxidase GPX4 for depletion of lipid peroxide and coenzyme Q oxidoreductase FSP1 and mitochondrial dihydrooratic acid dehydrogenase (DHODH) for neutralization of the lipid peroxide free radical (Bersuker et al., 2019; Mao et al., 2021) (Figure 2A). Hence, lipoperoxides cannot be excessively aggregated to disrupt the integrity of the plasma membrane. Due to the prevalence of the conserved cytochromes, LOXs, and other popular oxidoreductases and dehydrogenases in plants, the induced ferroptosis-like cell death may contribute to immune responses in plants upon biotic stresses (Figure 2C) (Distéfano et al., 2017; Dangol et al., 2019; Distéfano et al., 2021). Also, it has been reported that iron-dependent death regulates conidiospore development of pathogen fungi ——*Magnaporthe grisea* (Shen et al., 2020). The mechanistic insights into inhibition of ferroptosis in plants remain to be further elucidated by biochemical and genetic analyses.

### CNL Resistosome and Helper Nod-Like Receptors Mediate Ca^2+^ Influx Required for Programmed Cell Death in Plant Cells

The LRR-NBS domains from R proteins are largely similar to the LRR-NACHT domain in the inflammatory NOD-like receptor protein 3 (NLRP3) for caspase-1 activation. The *N*-terminal domains of R proteins can be divided into three categories: TIR-NBS-LRR (TNLs), CC-NBS-LRR (CNLs), and CC_R_-NB-LRR (RNLs). The three subfamilies are collectively referred to as Nod-like receptors (NLRs). NLRs evolved from a common primordial prokaryotic adenosine triphosphatase (ATPase), which is classified into two distinct derivatives: NACHT and NB-ARC type NBDs. The NB-ARC type is found in plant NLRs and NACHT in animal NLRs (Jones et al., 2016). Animal NLRs with cognate ligands can oligomerize into wheel-like complexes as inflammasomes upon stimuli (Hu et al., 2015). Similarly, the *Arabidopsis* ZAR1 protein (a CNL) initially confers resistance to *P. syringae* carrying the effector protein HopZ1a and sensitizes the alteration of the host sensory protein PBS1-LIKE 2 (PBL2) upon *Xanthomonas campestris* pv. *campestris* (Xcc) effector AvrAC (Bi and Zhou, 2021). The cryo-EM structures of the ZAR1 resistosome in resting and activated states were reported. ZAR1 interacts with the plant protein pseudokinase RKS1 (a receptor-like cytoplasmic kinase (RLCK), belonging to the RLCK-XII subfamily) and remained at the resting state. Upon infection, AvrAC uridylates PBS1-like protein 2 (a member of RLCK-VII subfamily) to generate PBL2^UMP^, which is recruited to the ZAR1-RKS1 complex to form the ZA1-RKS1- PBL2^UMP^ complex in a primed state lacking ATP or dATP (Wang et al., 2019a; Wang et al., 2019b). PBL2^UMP^ binding activates the nucleotide exchange factor activity of RKS1. Once activated, RKS1 facilitates ADP release from ZAR1 by inducing conformation changes in the NBD domain of ZAR, which enables ZAR1 to go through the fold switch of its CC domain and leads to the formation of a pentameric ZA1-RKS1- PBL2^UMP^ structure (Wang et al., 2019a) (Figure 2C). The funnel-shaped architecture constituted by the *N*-terminal alpha-helices of ZAR1 in the resistosome promotes ZAR1 integration into the plasma membrane. It may perturb the membrane integrity or ionic homeostasis. Subsequent studies showed that this funnel-like structure of helices in the N-terminal region is featured by a Ca^2+^ channel, being responsible for the Ca^2+^ influx, which is required for resistance (Bi et al., 2021). It is reminiscent that the activation of the inflammasome NLRP3 requires the serine/threonine kinase NEK7, and NLRP3-NEK7 modules constitute a disk-like structure (He et al., 2016; Sharif et al., 2019). This indicated the convergence on activation strategies of diverse varieties of NLRs. NLRs from animals and plants are all capable of being activated by kinase(s), which may possess nucleotide exchange activities and elicit the allosteric effect of NLRs to oligomerize into active resistosomes (Wang et al., 2019a).

TNLs RPP1 and roq1 recognize the bacterial effector ATR1 and *Xanthomonas* effector RPP1 of oomycetes, respectively. The direct binding of ATR1/RPP1 to a *C*-terminal jelly roll/Ig-like domain and LRR domain leads to induce the tetrameric assembly. The two catalytic centers of NADase are composed of asymmetric homodimers in tetrameric TIR domains, which define the formation of active holoenzyme (Martin et al., 2020; Ma et al., 2020). TNLs and CNLs directly or indirectly sensitize pathogen effectors. But RNLs are not conferred to perceive microbial avirulent factors but acts downstream of TNL-mediated signaling. Therefore, it is called helper NLR, which contains two types: NRG1 and ADR1. EDS1 and PAD4 are plant effector proteins with lipase-like domains, which aggregate to promote cell death. Additionally, NRG1 involves the downstream cascade of TNL-mediated cell death. Structural data and electrophysiological experiments also corroborated that the self-activating mutant of NRG1 is a calcium-permeable but non-selective cation channel. *Arabidopsis* NRG1 CC_R_ was structurally similar to pseudokinase MLKL as a cationic channel causing cell necrosis in animals (Jacob et al., 2021) (Figure 2C). Both self-activating mutants expressing NRG1 and ADR1 in tobacco and human cancer cells can give rise to cell death. And, the death phenotype depends on plasma membrane localization of helper NLRs and the Ca^2+^influx (Jacob et al., 2021). Collectively, the activation of NLR in CNL and TNL resistosomes both converge on the Ca^2+^ influx–mediated cation channels formed by NLRs. We have not yet understood how to trigger plant cell death after inducing the Ca^2+^ influx. According to previous reports, the appearance of HR required the plasma membrane fusion with a plant vacuole, in which cysteine proteases (including vacuolar processing enzymes (VPEs), and/or RD19) are required for cytolysis (Hatsugai et al., 2004; Bernoux et al., 2008).

### TIR as NADase for Producing Cyclic ADPR Essential for TNL-Mediated Death Signal Cascading and Activated Myd88-5 Inducing Neuron Death

**FIGURE 3:**
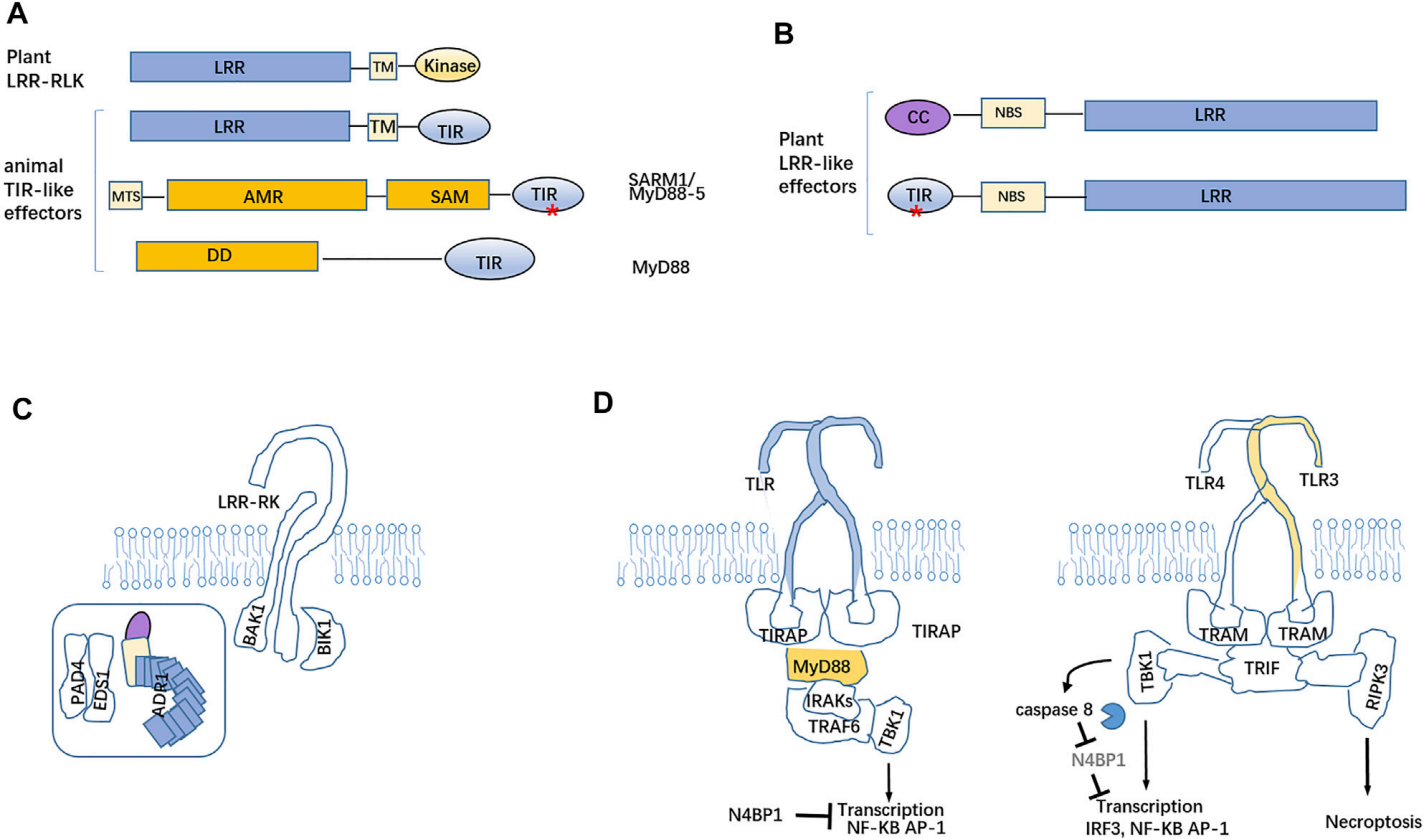
Mutual potentiation and regulation of TLR-mediated immunity and NLR-mediated immunity in plants and animals. **(A)** LRR-RLK (receptor-like kinase) confer to pattern-triggered immunity, functionally similar to animal Toll-like on the cell surface. SARM1 has a mitochondria-targeting signal (MTS), AMR, SAM, and C-terminal TIR domain, where there being conserved Asp/Glu residues, which endow the NADase activity. **(B)** Cytosolic CNL and TNL are composed of NBS and LRR domains with a distinct *N*-terminal region (coiled coil or TIR domain). **(C)** The PAD4-EDS1 complex and ADR1 together locate the inner leaflet of the plasma membrane and enhance PRR (LRR-RK)- and RLCK (BIK1)-dependent PTI. **(D)** Cytosolic toll and interleukin 1 receptor (TIR) of TLR4 can interplay with the TIR domain(s) of four adaptor molecules MyD88, TIRAP, TRIF, and TRAM to transmit the cascade reaction and promote the secretion of inflammatory factors and interferons. The capase-8 substrate, N4BP, act as a suppressor of cytokine responses. Asterisk (*) represents the catalytic activities of TIR domains.

The plant TNLs RPP1 and ROQ1 recognize their respective cognate effector ATR1. The complexes both individually oligomerize into tetramers, and the tetrameric wheel-like resistosomes have NADase holoenzyme activity through adjacent TIR–TIR close contact. The oligomerization of the TIR-domain is essential for its NAD^+^-catalyzing activity for variant cADPR (v-cADPR) production (Wan et al., 2019). TIR domains unlike other effector domains (such as Bcl-2, MLKL, and gasdermin) directly target the plasma membrane and perceive and/or amplify signals to trigger downstream immune responses (Figure 2D). TIR domains from plant TNLs have NADase activities versus TLR-containing TIR domains devoid of catalytic activities and are paralogous to the TIR domain of SARM1 protein, namely MyD88-5 (Figures 3A,B). MyD88 is an adaptor molecule involved in the signaling through the IL-1R and TLR families and is essential for the response to IL-1, IL-18, LPS, and many other bacterial cell-wall components. Otherwise, SARM1/MyD88-5 is the critical mediator of axon degeneration upon energy deficit (Jiang S et al., 2020).

The cryo-EM structure of full-length human SARM1 revealed that it bound NAD^+^ constitutes, an octamer in its inactive state, which inhibit its TIR NADase activity under high NAD^+^ levels (Bratkowski et al., 2020; Jiang Y et al., 2020; Sporny et al., 2020). The human SARM1 with a nicotinamide mononucleotide (NMN) octamer undergoes a conformational change disrupting NAD^+^binding sites of the ARM domains to enable TIR–TIR dimerization. NAD^+^deficit upon severe damages of the mitochondria from axons may result in SARM1 TIR-TIR associations and then produce cADPR to trigger axon death (Jiang Y et al., 2020). The ectopic expression of the TIR domain of human SARM1 in tobacco for cADPR generation may trigger cell death independent of EDS1 (Horsefield et al., 2019). The amount of cADPR dramatically increases upon accumulation of the senescence-related phytohormone ABA in plants and ultimately elicits cell death (Wu et al., 1997). It should be presumed that cADPR as a candidate common second messenger conveys death signal to the cascading pathways in plants and animals. In bacteria, cyclic oligonucleotide sensor(s) conjugated with effectors (such as lipase or transmembrane protein or other effectors) triggers enough bacterial cell disruption in response to phage invasion and results in the abortive infection (Severin et al., 2018; Morehouse et al., 2020). The infected bacterial cells commit suicide prior to the performance of the phage replication cycle. This strategy eliminates infected cells from the bacterial community and protects the bacterial population from a phagic epidemic (Hampton et al., 2020). Some bacteria exploit TIR-STING fusion protein to inhibit phage infections (Cohen et al., 2019; Eaglesham et al., 2019). Bacterial STING coupling cyclic dinucleotide recognition forms filaments to drive TIR oligomerization for cADPR production (Morehouse et al., 2020). This strategy is used to remove infected cells from the bacterial community and protect the population from a phage epidemic.

### Plant Immune Response Coordinated by PTI and ETI Versus TLR- and Nod-Like Receptor-Mediated Immune Responses in Animals

Upon pathogen infection, plants utilize cell-surface pattern-recognition receptors (PRRs) to rapidly recognize pathogen/damage-associated molecular patterns (PAMPs/DAMPs) and then bind and phosphorylate co-receptors—receptor-like cytoplasmic kinases (RLCKs) and phosphorylated RLCKs sequentially activate MAPK cascade signaling, Ca^2+^-dependent protein kinases (CDPKs), and reactive oxygen species (ROS) burst (Tang et al., 2017; Liang and Zhou, 2018). For examples, the NO burst was induced in *Arabidopsis* suspension cells in response to bacterial LPS. LPS treatment not only induces the expression of *Arabidopsis* NO synthase (AtNOS1) but also activates the defense genes (Zeidler et al., 2004). Sequentially, the *Arabidopsis* LPS receptor was identified to be lectin S-domain-1 receptor–like kinase LORE (Ranf et al., 2015). *AtNOS1*-deficient and LPS-insensitive *LORE* mutants are hypersusceptible to the pathogen *Pseudomonas syringae* (Zeidler et al., 2004; Ranf et al., 2015). Plant LysM domain proteins have been widely implicated in the recognition of GlcNAc-containing glycans. CERK1, a lysin-motif (LysM) receptor kinase (LYK) can recognize fungal MAMP chitin (Miya et al., 2007). LYK5 and LYK4 are also identified to be components of a tripartite chitin receptor complex (Cao et al., 2014; Xue et al., 2019). The glycosylphosphatidylinositol-anchored LysM proteins (LYM1 and LYM3) sense PGNs (Willmann et al., 2011). LRR-RK MIK2 recognizes multiple plant endogenous peptides of SCOOP family members, leading to a series of PTI responses, including the cytosolic Ca^2+^ influx, ROS burst, MAPK activation, ethylene production, and defense-related gene expression (Hou et al., 2021; Rhodes et al., 2021). The LRR receptor kinase HPCA1(other name CARD1) as the receptor(s) of DAMPs hydrogen peroxide and quinone perceives H_2_O_2_ and host-derived quinone DMBQ to activate the Ca^2+^ influx and MAPK pathway (Laohavisit et al., 2020; Wu et al., 2020).

These responses are collectively called pattern-triggered immunity (PTI), which can impede further invasion at the pre-infection phase. But, successful pathogens exploit secreted effectors dedicates to pathogen virulence to intervene with PTI; this leads to effector-triggered susceptibility (ETS); then, the host continuously evolves the novel NB-LRR protein(s) to specifically recognize/sequester pathogen effector(s), inducing effector-triggered immunity (ETI) (Jones and Dangl, 2006). R protein-mediated ETI accelerates and amplifies immune responses, leading to resistance against diseases, usually, a hypersensitive cell death response (HR) at infection sites (Jones and Dangl, 2006). Certainly, ETI has two-branched responses: promotion of the resistance-related gene expression and HR. Recent advances confirmed that ETI also robustly boosted the expression of PTI-involved genes; meanwhile, the sole activation of NLR-mediated resistance in absence of PTI is insufficient to safeguard the host against bacterial infections (Yuan et al., 2021). PTI can enhance resistance from PTI. The PTI and ETI comprising the two-tiered plant immune system that monitors threats from pathogens are not separable and collaboratively contribute to the reciprocal enhancement of plant immunity (Ngou et al., 2021; Pruitt et al., 2021; Yuan et al., 2021; Tian et al., 2021). HR as one canonical type of PCD in plants is beneficial to the host likely by eliminating the intracellular niche for proliferation of certain pathogens. Furthermore, the resulting cellular debris coordinates a systemic immune response to promote the resolution of infection. As known, HR cannot represent the whole resistance in plants. Reprogramming of immune genes enforces resistance. The ADR1-EDS1-PAD4 module can be polymerized with the complex formed by LRR-RP-SOBIR1 and PBL31 of the RLCK family to form a supramolecular complex, which not only binds the inner leaflet of the plasma membrane to mediate the response of PTI but ADR1 is contributive to TNL-directed resistance signaling involved in reprogrammed transcription of pathogenesis-responsive genes (Pruitt et al., 2021) (Figure 3C).

Likewise, the Toll-like receptor (TLR) families in animals are phylogenetically conserved mediators of innate immunity and are responsible for microbial recognition on cellular surfaces. TLRs consist of a large family with extracellular/ectodomain leucine-rich repeats (LRRs) and a cytoplasmic Toll/interleukin (IL)-1 receptor (TIR) homology domain (Fitzgerald and Kagan, 2020). TLRs occupy the plasma membranes and detect the microbial conserved components present on the host cell surface. TLRs sensitize peptidoglycan (TLR2), dsRNA (TLR3), lipopolysaccharide (LPS) (TLR4), flagellin (TLR5), unmethylated CpG DNAs (TLR9), and other PAMPs. In virtue of the unraveled extracellular receptors for molecular patterns in plants, more diverse motifs present in the extracellular space are used for sensing various molecular patterns including MAMPs and/or other stimuli. Moreover, the commonalities between receptors in plants and animals are single-pass transmembrane proteins which recruit cytosolic kinase(s) to activate phosphorylation-cascading pathway(s) and induce the expression of stress-related genes. In virtue of alien dsRNA or ssDNA being conserved signatures, plant hosts should license PRRs to perceive the immune signal, although the potent extracellular dsDNA/RNA receptors located at the plant cell surface are yet to be validated.

Upon recognition of PAMPs, the cytoplasmic TIR domains of dimerized TLRs located at the plasma membrane recruit TIR-containing TIRAP and Myd88 to assembly Myddosome containing TRAF6, which functions to stimulate TBK1 to drive IKK- and MAPK-dependent transcription and cytokine releases (Fitzgerald and Kagan, 2020). On endosomes, TLR4 and TLR3 are capable of engaging a SMOC called the triffosome. TRIF is present in this complex. TRIF encompasses a pLxIS motif that increases the TBK1-regulated gene expression and an RHIM domain to promote RIPK3-mediated necroptosis (He et al., 2011) (Figure 3D). Under certain conditions, the TLR3 and TLR4 pair activates caspase-8 through the adaptor TRIF, but generally TLR3/4 signaling does elicit apoptosis. Caspase-8 cleaves N4BP1, which inhibits cytokine responses and suppression of the LPS-stimulated gene expression (Gitlin et al., 2020) (Figure 3D). Functionally, TLRs from animals may be regarded as the counterpart of LRR-RLKs involved in PTI in plants. The cytoplasmic NOD-like receptors (NLRs) monitored the intracellular environment for an alternative sign upon pathogen infection and then fulfill the assembly of inflammasomes. In parallel, plants have evolved intracellular R proteins to intercept the activities of effector proteins that are delivered inside the host cell and activate defenses, complementing the ETI, which resembles inflammasome-mediated innate immunity in animals. Despite many breakthroughs on the understandings of PTI and ETI from plants and the proposed conceptual resistosome in parallel with the inflammasome, the direct executioners of HR in plants need to be further investigated and explored by the state-of-the-art and maneuverable technology.

### Outlook

Mammalian intrinsic apoptosis is marked by the mitochondrial membrane rupture, which resulted from Bax/Bak oligomerization in mitochondrial membranes. The mitochondria branches within alphaproteobacteria. And, the pore-forming domains of bacterial toxins such as colicins A1 and E1 and diphtheria toxin structurally are similar to mitochondrial Bcl-2 (Kelekar and Thompson, 1998). Like bacterial toxins, Bcl-2, Bcl-xL, and Bax can insert into synthetic lipid vesicles and planar lipid bilayers and form ion-conducting channels (Schendel et al., 1997). This suggested that the Bcl-2 family responsible for mitochondrial “suicide” might have ancestral origins from bacteria. Why PCD in host cells requires that semi-parasitic mitochondria commit self-death in advance? Apoptosomes sense oxidation–reduction potential to activate major initiator caspase-9 for intrinsic apoptosis. Indeed, pyroptosis is also concomitant with damaged mitochondria. The elevated mitochondrial ROS promote gasdermin pore formation for pyroptosis.

Similarly, the plant NLRs activate downstream immune responses, which escalate the expression level of crucial immune proteins, such as BIK1 and NADPH oxidase RBOHD. A plethora of heme-containing RBOHD located in the plasma membrane boost ROS burst (Yuan et al., 2021). R protein-mediated HR coincides with the coexistence of Fe^2+^ and ROS, which both being requisite for ferroptosis. As aforementioned, NLRs (CC- and CC_R_-types) in plants possess similar structural features of inflammatory NLRs and necroptotic MLKL in animals. These appeared to be indicative of HR in plants as PCD was featured by the chimeric/promiscuous combination of pyroptotic, necroptotic, and ferroptotic forms. According to the above descriptions and comparisons, the PRRs (such as LRR-RK) and R proteins (TNLs and CNLs with LRR motifs) from plants structurally and evolutionarily correspond to TLRs and NLRs (both comprising LRR motifs) in animals. Evidently, the common superhelical conformation of distinct LRR motifs is selected to recognize the diverse molecular patterns derived from pathogens or hosts in plants and animals. Second, ROS act as common inducers for PCD in plants and animals. Third, the serious damage/disruption of biomembranes (the plasma membrane and/or mitochondria/plastid membrane) is essential for PCD in plants and animals. Considering the extreme diversity of bacteriophage species and their tremendous amounts, bacteria hosts exert utmost efforts on the development of immune arsenal including caspase-like,TIR-like, cGAS-like, STING-like, and lipase-like domains shared by prokaryotes and eukaryotes (Figure 2B). Based on the subsequent discoveries of the primordial signature of bacterial immune proteins orthologous to counterparts in animals, there are grounds to believe that plants have uncharacterized immune effectors which might contribute to conserved PCDs and which might be evolved from the common ancestors/progenitors shared by bacteria and animals.

Relative to the clear mechanistic insights into animal PCDs, in plant PCDs there are many important unsettled problems: 1) the final executioner of plant HR death upon infections; 2) the exact substrates of cysteine proteases or other proteases involved in PCD; 3) the role of the Ca^2+^ influx. 4) the definite signalings conjugated with the second messengers; 5) the linkage of various types of plant PCDs upon infections; 6) the necessary roles of lipase-like activities in plant HR. Additionally, plant cells have rigid cell walls surrounding the plasma membrane. The question as to whether or not the cell death of plant cells required the degradation of cell wall components such as cellulose, hemicellulose, and pectin remains unknown. We should recapitulate the cellular states including transcriptional and translational profiles through single-cell multi-omic analyses. Collectively, these embodies that the uniform but diversity in all organism PCDs involved in immunity.
